# Expression of growth factors in buffalo ovarian tissue across different follicular developmental stages

**DOI:** 10.1007/s00404-025-08090-8

**Published:** 2025-06-21

**Authors:** Seham Samir Soliman, Marwa El-Sheikh, Dalia A. Taha, Karima A. Hamed, Wagdy K. B. Khalil

**Affiliations:** 1 Department of Animal Reproduction and Artificial Insemination, Veterinary Research Institute, National Research Centre (NRC), Dokki, Cairo, 12622 Egypt; 2 Department of Microbial Biotechnology, Biotechnology Research Institute, National Research Centre (NRC), Dokki, Cairo, 12622 Egypt; 3 Department of Cell Biology, Biotechnology Research Institute, National Research Centre (NRC), Dokki, Cairo, 12622 Egypt

**Keywords:** Follicle development, Buffalo, Ovarian tissue, mRNA transcription, Oxidative stress

## Abstract

**Background:**

In assisted reproduction, poor ovarian response to stimulation negatively affects oocyte yield and is influenced by genetic factors.

**Objective:**

This study aimed to quantify the mRNA expression of key growth markers (BMP15, GDF9, OCT4, and FGFR2) in ovarian tissue according to the developmental stages of the follicle.

**Methods:**

Samples were collected from ovarian tissue. Gene expression levels were analyzed using RT-qPCR. In addition, ELISA was used to measure the concentrations of catalase (CAT), glutathione peroxidase (GPx), and superoxide dismutase (SOD), along with reactive oxygen species (ROS) and malondialdehyde (MDA) as oxidative stress markers.

**Results:**

OCT4 expression was similar in preantral and small follicles but significantly upregulated in medium and large follicles. GDF9 expression and SOD activity were highest in small follicles (*P* < 0.05). BMP15 levels were significantly elevated in small and medium follicles compared to preantral follicles but remained unchanged in large follicles (*P* < 0.05). FGFR2 expression increased progressively with follicle size (*P* < 0.05). GPx activity was directly proportional to follicle size, with the lowest levels in preantral follicles. Conversely, ROS, MDA, and CAT concentrations decreased as follicle size increased.

**Conclusion:**

These findings provide insights into the molecular regulation of follicular development in buffalo, which could aid in improving reproductive efficiency in assisted reproduction programs.

## What does this study add to the clinical work?

**Table:** 

Follicular development in buffalo is regulated by stage-specific expression of growth-related genes (BMP15, GDF9, OCT4, FGFR2) and oxidative stress markers. Smaller follicles show higher oxidative stress and distinct gene expression profiles, while larger follicles exhibit enhanced antioxidant activity and upregulation of key developmental genes. These molecular patterns may inform strategies to improve ovarian response and reproductive outcomes in assisted reproduction.	

## Introduction

Follicular development in the mammalian ovary is a highly complex and tightly regulated process involving numerous cellular events, including differentiation, proliferation, and growth. The ovarian follicle serves as the fundamental functional unit that undergoes sequential developmental stages before ovulation. Folliculogenesis begins with the formation of the preantral follicle, which is characterized by the differentiation and growth of both the oocyte and surrounding follicular cells. The initial stage of this process involves the formation of primordial follicles, which constitute the ovarian follicular reserve. This reserve is established in all mammalian ovaries and plays a crucial role in determining the reproductive potential of females throughout their lifetime. As folliculogenesis progresses, primordial follicles develop into primary and subsequently secondary follicles. With the onset of puberty, follicular development becomes cyclic, leading to the formation of antral follicles each month in preparation for ovulation [1].

The oocyte serves as the central regulator of ovarian function, playing a pivotal role in follicular development. Among the key oocyte-derived factors, bone morphogenetic protein 15 (BMP15) and growth differentiation factor 9 (GDF9) have been identified as crucial regulators of this process [2, 3]. These factors are highly expressed in cumulus and granulosa cells and are involved in various developmental stages, including oocyte maturation, fertilization, embryonic quality, and overall follicular growth [4, 5]. Through autocrine and paracrine signaling mechanisms, BMP15 and GDF9 orchestrate the growth, differentiation, and functionality of oocytes and their surrounding granulosa and theca cells. Moreover, their roles extend to reproductive health, influencing conditions such as primary ovarian insufficiency, polycystic ovary syndrome (PCOS), and endometriosis. Understanding the molecular and cellular mechanisms of BMP15 and GDF9 is essential for advancing fertility research and developing therapeutic strategies for reproductive disorders [6, 7].

Octamer-binding transcription factor 4 (OCT4) plays a crucial role as a cellular regulator in various biological processes, including proliferation and development. Notably, OCT4 is highly expressed in oocytes within primary follicles and is significantly influenced by gonadotropins, which regulate the growth of oocytes, granulosa cells, and embryos [8–11]. Previous studies have demonstrated that OCT4 precisely modulates follicular development in vitro [10]. Furthermore, research investigating the effects of OCT4 overexpression on the transfection of porcine stromal and ovarian cells revealed that OCT4 induction enhances the differentiation and formation of ovarian follicles in vivo while promoting the production of oocyte-like cells in vitro [13].

Fibroblast growth factor receptor 2 (FGFR2) is a membrane-bound tyrosine kinase receptor that belongs to the fibroblast growth factor (FGF) family. This family comprises various genes that mediate essential cellular signaling pathways involved in development, growth, survival, organogenesis, proliferation, and differentiation. These roles have been demonstrated in oocytes, ovarian tissues, and embryos [14, 15]. FGFs are expressed in oocytes, embryos, and ovarian follicles during different developmental stages, including in bovine species. FGFR2 has been identified as a key regulator in promoting pre-implantation embryo development and supporting granulosa cell survival [14, 17, 18].

Furthermore, FGF2 has been shown to enhance the survival rate and quality of oocytes, as well as cryopreserved or transplanted ovarian tissues in mice [16]. In vitro studies have also demonstrated that FGF2 supplementation promotes ovarian follicular development, growth, and survival [20]. In humans, FGF2 has been reported to support the survival and development of early ovarian follicles [20]. While previous studies have explored the expression of FGFs, the OCT4 marker, and key oocyte-secreted factors in oocytes, cumulus–oocyte complexes, and embryos, as well as the importance of growth markers in the promotion of in vitro growth of ovarian follicles, there remains a need to further investigate and compare their roles in regulating follicular development. These applications might be helpful to improve follicle health under cancer conditions and long-term storage conditions of ovarian tissue [21, 22]. Despite extensive research on growth factors and their role in ovarian follicle development in many species, limited information is available on their specific roles in buffalo [24, 25]. Buffalo, an economically important species in many regions, exhibits unique reproductive physiology, necessitating species-specific studies. Understanding the impact of growth factors on buffalo ovarian follicle development is crucial for optimizing IVF protocols, improving oocyte quality, and enhancing overall reproductive outcomes.

In the present study, we hypothesize that the fertility markers OCT4, FGFR2, GDF9, and BMP15 play a crucial role in regulating folliculogenesis at various stages of ovarian follicle development. Our objective was to examine the mRNA expression patterns of these markers in ovarian tissue and compare their transcriptional levels across different follicle stages, including preantral, small, medium, and large follicles. In addition, we assessed the activity of key antioxidant enzymes—superoxide dismutase (SOD), glutathione peroxidase (GPx), and catalase (CAT), as well as the levels of reactive oxygen species (ROS) and malondialdehyde (MDA) during both the early and late stages of follicular maturation. Both biochemical and molecular investigations will help in the understanding of the crucial role of the selected growth markers in the regulation of folliculogenesis.

## Materials and methods

### Ovaries collection and follicles classification

From the local abattoir, the slaughtered buffalo’s ovaries (ten for each group) with different sizes of follicles were collected. All the animals were cycling. Samples were transported on ice to the laboratory within 2 h of collection. Preantral follicles (with more than one layer of granulosa cells) were considered as control, only healthy follicles were used in the current study and follicles with non-transparent fluid and non-well-organized arrangements were excluded. Experimental groups were classified based on their size into three categories: small follicles (SF < 3 mm in diameter), medium follicles (MF = 3–6 mm), and large follicles (LF > 7 mm) [26] and the control (preantral follicles), *n* = 50/ovary represent an earlier stage of follicular development, allowing for a comparison with more advanced follicular stages to identify developmental changes. The experimental work was conducted during the period from January to March 2024.

### The isolation of preantral follicles from water buffalo

Both ovaries with and without corpus luteum were used in the study. The ovarian cortex was sliced using a surgical blade to 3 mm or less in size in a plastic Petri dish. Preantral follicles were isolated from ovarian pieces by microdissection; the ovaries were cut into fragments using a scalpel blade and were completely fragmented with a pair of 12 cm straight-bladed scissors. Fragments were transferred into a 15- or 50-mL falcon tube containing 10–20 mL phosphate-buffered saline (PBS), and then homogenized using a homogenizer, then vigorously pipetted using a Pasteur pipette for 2–3 min. This homogenate was filtered through a 500 µm nylon mesh adapted to a 50 mL Falcon tube. The homogenate was allowed to settle for 10 min at room temperature. Then the precipitate was transferred into a 10 cm culture plate containing 10 mL PBS and 4 mg/mL bovine serum, which was counted under a stereomicroscope. Preantral follicles ranging from 250 to 400 µm in diameter were selected. For each isolation operation, a total of 3–4 ovaries were utilized. The preantral follicles collected from each ovary were used to obtain the necessary amount of total RNA.

### Total extraction of RNA, cDNA synthesis to qRT-PCR

From the ovarian tissue samples derived from the different developmental stages of follicles, including SF, MF, and LF, we extracted the total RNA. The extraction of total RNA was performed using the TRI Sure Chloroform kit and according to the manufacturer’s guidelines. One-step RT-PCR has been used in the current study and cDNA synthesis was performed according to the following conditions: 45 °C for 20 min, a pre-denaturation step, and then 35 PCR cycles consisting of denaturation for 10 s (95 °C), annealing for 20 s (59 °C), extension for 30 s (72 °C), and final extension for 7 min (72 °C). Purity of total RNA was assessed by the 260/280 nm ratio (between 1.8 and 2.1). Aliquots were used immediately for reverse transcription (RT), otherwise stored at − 80 °C. Approximately 5 μg of the total RNA was used to synthesize the cDNA.

cDNA from different categories of buffalo follicles was used as templates for qRT-PCR. The follicular cDNA was determined using the Step One Real-Time PCR System (Applied Biosystems; Thermo Fisher Scientific, Waltham, MA, USA). The PCR reactions were set up in 25 μL reaction mixtures that had 12.5 μL of SYBR® Premix Ex TaqTM (made by TaKaRa Biotech. Co. Ltd.). The mixture consists of 0.5 µL of a 0.2 µM concentration of sense primer, 0.5 µL of antisense primer, distilled water, and the cDNA template. The reaction program was divided into three distinct steps. The initial stage of denaturation is a duration of 3 min. The second stage involved a total of 40 cycles, as follows: (a) heating at 95.0 °C for 15 s, (b) cooling at 55.0 °C for 30 s, and (c) heating at 72.0 °C for 30 s. Lastly, it began at a temperature of 60.0 °C and subsequently rose by approximately 0.5 °C every 10 s until reaching 95.0 °C. Every experiment incorporated a control group consisting of distilled water. The primers for growth and maturation-related genes, including OCT4, BMP15, GDF9, and FGFR2, were constructed and listed in Table 1. For each mRNA, gene analysis was repeated five times. Relative quantification of the selected genes to the reference was obtained using the 2^*−*ΔΔCT^ technique [27].

**Table 1:** Primers used for RT-qPCR analysis of buffalo’s follicles

Gene	Primer sequence	Accession No.
OCT4	F: CAG CCA AAC GAC TAT CTG CC R: CTC GTC CGC TTT CTC TTT CG	EU926737.1
BMP15	F: TGG GGC TGA CTT CAT ACT GG R: TTC CTT CCA CCC ACT GTC TC	XM_055562914.1
GDF9	F: AGA CCA TCC TGT GTA CCT GC R: TTT ACA CTG GCC AGG ACA CT	XM_055553605.1
FGFR2	F: ATA TAC GTG CTT GGC GGG TA R: AAG ATG ACC GTC ACC ACC AT	XM_055559915.1
GAPDH	F: AGA TGG TGA AGG TCG GAG TG R: TGG AAG ATG GTG ATG GCC TT	XM_055569036.1

*F* forward, *R* reverse

### Measurement of antioxidant enzyme activity of superoxide dismutase (SOD)

The estimation of SOD was conducted using the methodology developed by Madesh and Balasubramanian [28]. The assay mixture, containing 90 µL of freshly produced pyrogallol solution, was combined with MTT (1.25 mM), PBS (pH 7.4), and homogenized follicle tissues. Next, mixtures were incubated for 5 min, and the reaction was cut using around 1 mL of dimethyl sulfoxide (DMSO). The tubes were agitated, and the optical density was determined at a wavelength of 570 nm using a double-beam UV–Vis spectrophotometer (V-630 UV–Vis spectrophotometer from Jasco, Manchester M1 4ET, UK). Simultaneously, a reagent blank was generated by substituting the sample with distilled water in equal volumes. The quantity of superoxide generated was determined by utilizing MTT formazan E570 molar extinction coefficient, which is 17,000/M/cm when the pH is equal to 7.4. The percentage of inhibition caused by SOD was calculated based on the decrease in MTT color formation as compared to the MTT formazan produced in SOD absence and was 100%.

### Estimation of antioxidant enzyme activity of catalase (CAT)

The measurement of CAT activity was conducted in lysates of homogenized follicular tissues. We combined approximately 5 mL of the lysate with 2000 μL of phosphate buffer and 1000 μL of a 30 mM hydrogen peroxide (H₂O₂) solution in a cuvette. We targeted the spectrophotometer to analyze the CAT activity at a wavelength of 240 nm for a duration of 1 min. The catalase activity was determined using the molar extinction H_2_O_2_ coefficient. The activity corresponds to the degradation of 1 mmol of H₂O₂ corresponding to 1 mL.

### Glutathione peroxidase (GPx) enzyme activity detection

GPx activity was measured using the idea that GPx facilitates the reaction between H_2_O_2_ and reduced glutathione (GSH), resulting in the formation of oxidized glutathione (GSSG) and water. The GPx activity measures the rate at which H₂O₂ oxidizes the compound GSH. The assay mixture comprised 0.2 mL of homogenized follicular tissue lysate, 1 mL of sodium phosphate buffer (pH 7.2), 1 mL of GSH (2 mM), 0.5 mL of sodium azide (0.01 M), and distilled water. We incubated the mixture for 5 min. A volume of 1 mL of H_2_O_2_ was subjected to the test mixture and allowed to incubate for a duration of 3 min at a temperature of 37 °C. Following the incubation period, we combined 1 mL of the reaction mixture with 4 mL of a precipitation solution containing meta-phosphoric acid. The mixture was centrifuged at 500*g* for 10 min. 2 mL of the resulting liquid settled at the top after centrifugation. The mixture was thoroughly mixed, and the amount of light absorbed was measured at a wavelength of 421 nm using a double-beam UV–Vis spectrophotometer. GPx was quantified as the reduction in log (GSH) by 0.001/min after subtracting the reduction in log (GSH per minute) caused by the non-enzymatic process.

### Malondialdehyde (MDA) assessment

MDA concentration was used to evaluate lipid peroxidation in homogenized follicular tissues. A TBA–TCA reagent, consisting of tri-Chloro acetic acid 15% (w/v), TBA 0.375% (w/v), and dissolved in HCl, was introduced to a 1 mL suspension of follicular cells. The combination was subjected to a process of heating in a water bath at its boiling point for a duration of 15 min. Following the cooling process, the suspension underwent centrifugation at a force of 500*g*. The liquid generated (the supernatant) was then separated. Supernatant absorbance was detected at a wavelength of 535 nm and using a double-beam spectrophotometer.

### Determination of the levels of intracellular reactive oxygen species (ROS)

This study utilized 2′,7′-DCFH-DA fluorescent dyes to determine the ROS level in the follicle cells using flow cytometry [29]. In summary, we resuspended percoll-washed follicular cells in 1 mL of PBS. They were then treated with DCFH-DA at 1.0 µM in the dark and at 37 °C for 15 min, followed by the addition of propidium iodide (PI) at a final concentration of 25 µg/mL. The follicular cells that had been labeled were rinsed with PBS and examined using a flow cytometer. We used the FACS Calibur for flow cytometric analysis. The fluorescence of DCFH-DA was measured, while the fluorescence of PI was measured using a long pass filter with a wavelength of 620 nm (FL-2). In each sample, a total of ten thousand follicular cells were obtained and examined at a rate ranging from 50 to 500 events per second. These cells were then further analyzed using Cell-Quest software from Becton Dickinson-Siteward.

### Statistical analysis

The mRNAs, antioxidant enzymes, MDA, and ROS levels in the buffalo’s follicles were analyzed using the procedure of General Linear Models (GLM) of the Statistical Analysis System (SAS), followed by the Scheffé test for the multiple comparisons between the experimental groups. Values were presented as mean values ± standard error of the mean (SEM), and the significant value was shown as *P* values < 0.05.

## Results

### Evaluation of gene expression of the selected candidate genes in buffalo’s follicles

**Fig. 1:**
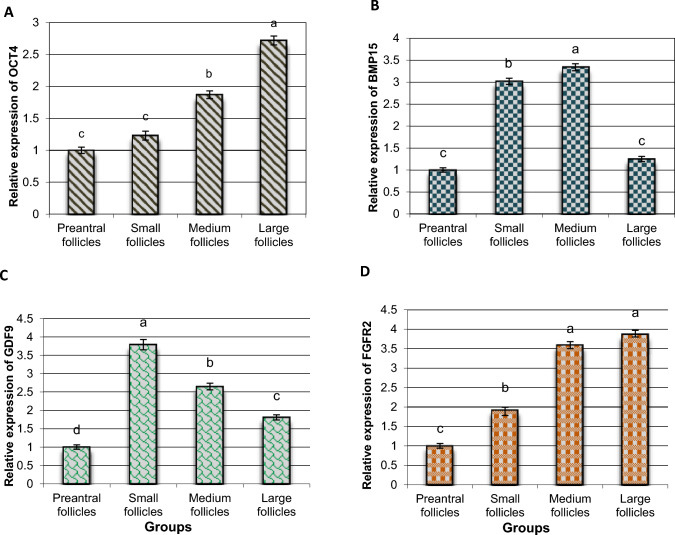
The expression of the *OCT4, BMP15, GDF9,* and *FGFR2* genes in different buffalo follicles. Values are presented as mean ± SEM. ^a,b,c,d^Statistically significant differences (*P* < 0.05)

Our results demonstrated that OCT4 expression levels in preantral and small follicles were relatively similar with low levels compared to medium and large follicles. The expression levels of OCT4 in medium and large follicles were increased significantly (*P* < 0.05) by 187 and 272%, respectively, compared with preantral follicles as the control group (Fig. 1A). Moreover, the expression levels of the OCT4 gene reached the highest levels (*P* < 0.05) at the large follicles in comparison with the control group and other follicle types.

In small and medium follicles, BMP15 was increased significantly by 302 and 334% compared with preantral follicles, while in large follicles, its expression was downregulated to similar levels as in preantral follicles (Fig. 1B). Also, there were clear differences in the amounts of GDF9 mRNA between the groups that were tested. The levels were highest in small follicles, which was significantly different (*P* < 0.01) from preantral and other tested follicles (Fig. 1C). Moreover, expression levels of the GDF9 gene in medium and large follicles were significantly upregulated by 379 and 265%, respectively, compared to those in preantral follicles.

In addition, checking the expression profile of the FGFR2 gene showed an induction in the FGFR2 mRNA levels in a follicle size-dependent manner (*P* < 0.05). The highest expression levels were detected in large follicles > medium > small > preantral follicles (Fig. 1D).

### Concentrations of different antioxidant enzymes in the developmental stages of buffalo’s follicles

**Fig. 2:**
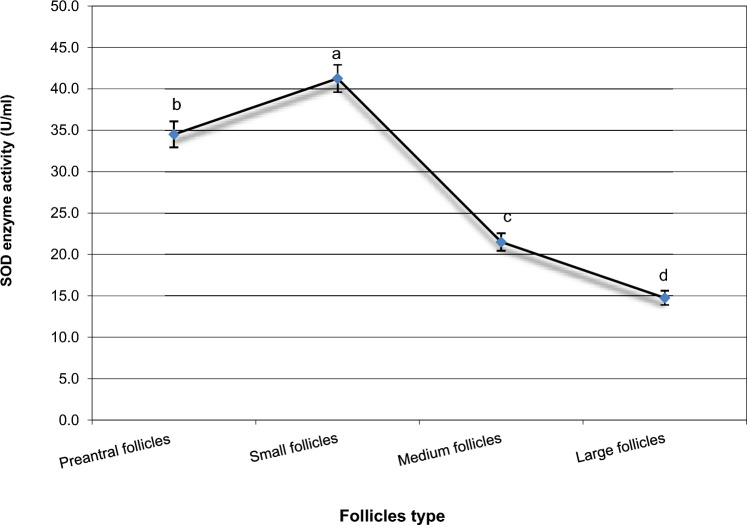
Intracellular SOD enzyme activities in different buffalo’s follicles. Mean ± SEM was selected to present the data. ^a,b,c^Statistically significant differences (*P* < 0.05)

We demonstrated a significant increase of SOD in small follicles as compared to preantral follicles, medium, and large follicles (Fig. 2). Moreover, SOD enzyme activity in preantral follicles was significantly higher than in medium and large follicles (*P* < 0.05). In addition, as seen in Fig. 2, medium follicles exhibited SOD enzyme activity more than those in large follicles.

**Fig. 3:**
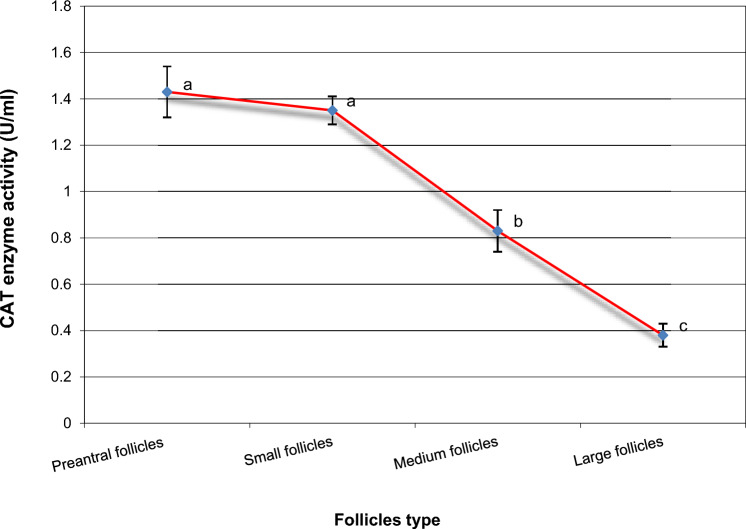
Intracellular CAT enzyme activities in different buffalo follicles. Mean ± SEM was selected to present the data. ^a,b,c^Statistically significant differences (*P* < 0.05)

Next, the activity of the CAT enzyme was checked, and in preantral follicles, the CAT levels were significantly higher than those in small, medium, and large follicles. Furthermore, the CAT enzyme activity in small follicles was significantly higher (*P* < 0.05) than those in medium and large follicles (Fig. 3). In addition, medium follicles exhibited CAT enzyme activities more than those in large follicles (Fig. 3).

**Fig. 4:**
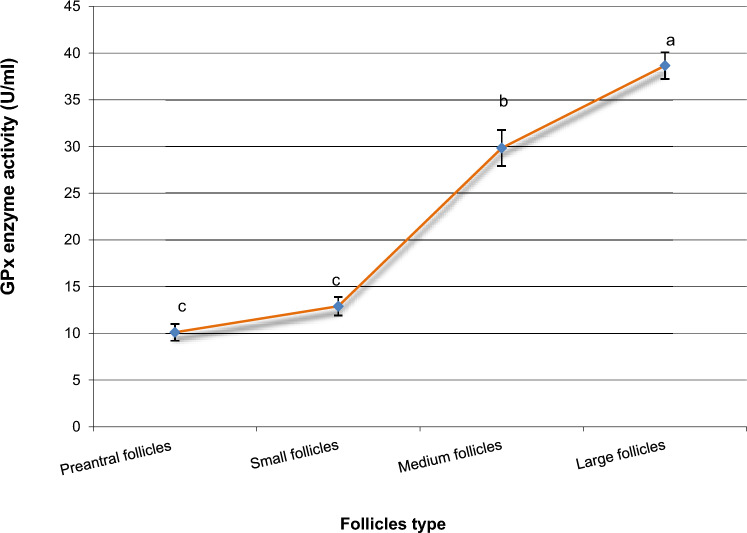
Intracellular GPx enzyme activities in different buffalo’s follicles. Mean ± SEM was selected to present the data. ^a,b,c^Statistically significant differences (*P* < 0.05)

Likewise, activity of GPx enzyme in large follicles showed a significant increase (P < 0.05) than those in preantral, small, and medium follicles (Fig. 4). GPx enzyme activity in medium follicles was significantly higher (*P* < 0.05) than those in preantral and small follicles (Fig. 4). However, small follicles exhibited GPx enzyme activities more than those in preantral follicles, although these levels did not reach to the significant difference (Fig. 4).

### Oxidative stress and MDA lipid peroxidation marker estimation during the different stages of buffalo’s follicles

Further, we estimated the intracellular levels of MDA and ROS in buffalo follicles. Our results demonstrated that MDA and ROS levels were significantly higher in preantral and small follicles compared to medium and large follicles (Table 2). However, MDA and ROS levels were increased in preantral follicles compared with the small follicles but without showing a significant difference. Moreover, MDA and ROS levels in medium follicles were significantly higher than the levels detected in large follicles (Table 2).

**Table 2:** MDA and intracellular ROS levels in different buffalo follicles

Follicles type	MDA (nmol/mL)	ROS (mean fluorescence intensity)
	Mean ± SEM	Mean ± SEM
Preantral follicles	2.18 ± 0.09^a^	49.92 ± 1.25^a^
Small follicles	1.97 ± 0.08^a^	47.30 ± 1.23^a^
Medium follicles	1.24 ± 0.04^b^	30.49 ± 1.14^b^
Large follicles	1.02 ± 0.06^c^	22.50 ± 1.17^c^

^a,b,c^Statistically significant difference (*P* < 0.05)

## Discussion

Ovarian folliculogenesis is a highly multifaceted process involving different mechanisms regulating cellular and molecular events essential for the development of competent oocyte [30]. The initial stage of this process involves the formation of primordial follicles to serve reproduction functionality of females [31]. The size of the follicle was reported to be associated with the rates of oocyte maturation and subsequent embryo development, making it as a useful indicator reflecting oocyte health, as well as the ART success rates [32]. In the current study, we investigated the influence of specific growth factors on buffalo ovarian follicle development, providing new insights into their regulatory roles. Our findings demonstrate that growth factors, such as GDF9 and BMP15, significantly influence follicular development, consistent with their known roles in other species, including cattle, sheep, and humans. However, our results offer a unique perspective on their function in buffalo, contributing to the limited understanding of reproductive physiology in this species. In the current study, follicles were categorized based on their diameter: those measuring ≤ 4 mm were classified as small, those between 4 and 6 mm as medium sized, and those ≥ 6 mm as large follicles [19]. Mammalian ovarian follicle development is a multifaceted process that involves the precise regulation of several biological events, including apoptosis, proliferation, and differentiation theca cell growth and migration towards the medulla [33, 34].

Samples have been used to investigate the mRNA expression of specific fertility markers involved in the regulation of oocyte and follicle development. Moreover, oxidative stress parameters have been assessed across various follicle developmental stages.

GDF9 and BMP15 are essential regulators involved in various critical aspects of fertility, oogenesis, and reproduction [2–4, 7]. These factors are predominantly expressed in both oocytes and cumulus cells, where they function as key regulators of ovarian quality and overall reproductive function [7]. In addition, GDF9 and BMP15 play an indispensable role in the development of the corpus luteum and follicular maturation, processes that are crucial for successful ovulation [5]. Disruptions or mutations in the genes encoding these regulatory factors have been linked to several fertility disorders, including endometriosis, chronic hormonal imbalances, and polycystic ovary syndrome [6, 7]. Moreover, the regulation of several biological events including apoptosis, proliferation, and differentiation, as well as cell growth and migration such as in theca cells have been addressed in different studies to be linked to follicle health and development. Also, the formation of a highly competent oocyte relies on a series of complex signaling pathways within the ovarian follicle [35]. Notably, the essential roles of oocyte-specific factors such as GDF9 and BMP15 have been well-documented in previous studies [2, 3]. In the present study, we observed a significant upregulation of BMP15 mRNA expression in small and medium follicles compared to the control group. Conversely, large follicles exhibited a marked decrease in BMP15 expression, reaching levels like those observed in preantral follicles (control group). Regarding GDF9, we found notable variations in its mRNA levels across the experimental groups. GDF9 expression was highest in small follicles, showing a significant increase (*P* < 0.05) when compared to preantral and other follicle stages.

Moreover, GDF9 expression was also markedly upregulated in medium and large follicles, surpassing the levels observed in preantral follicles (*P* < 0.05). Our findings align with previous studies investigating follicular development, where GDF9 treatment was shown to enhance the developmental capacity of follicles. Specifically, one study reported that follicles in the primary stage exhibited the highest developmental rates compared to follicles at later stages, in response to GDF9 treatment [36]. In addition, the expression levels of both GDF9 and BMP15 in follicular fluid have been reported to be decreased in younger populations with a lower prognosis for successful ART and embryo transfer treatments [37]. In contrast, another study demonstrated that GDF9 and BMP15 promote the in vitro development of human follicles, with GDF9 showing a particularly beneficial impact [38]. These findings align with our current results, further supporting the direct involvement of these markers in follicular development.

Our data reinforces the role of GDF9 in ovarian follicle development, as evidenced by its upregulation across different stages of follicular development, particularly when compared to preantral follicles. Although the difference in BMP15 expression between large and preantral follicles was not statistically significant, a marked increase in BMP15 expression was observed in both small and medium follicles relative to the control group. This highlights the importance of follicle size in regulating critical growth markers within ovarian tissue.

Previous studies have highlighted the role of fibroblast growth factors (FGFs) in promoting the transition from ovarian primordial follicles to primary follicles [39]. FGFs are known to regulate a variety of biological processes, including growth, development, proliferation, and differentiation, particularly in reproductive tissues, oocytes, and embryos across different species [14, 15]. In humans, FGFs have been shown to support the survival and development of ovarian follicles in vitro [20]. Our results also indicate that the expression of the FGFR2 gene is upregulated in a manner dependent on follicle size (*P* < 0.05), with the highest expression observed in larger follicles.

On the other hand, OCT4, a key transcription factor, regulates various molecular cascades that enhance growth and differentiation. It has been shown to be expressed in oocytes, where it plays a crucial role in meiosis regulation and the maturation process. OCT4 is also present in the granulosa cells of early-stage and growing follicles, with studies suggesting its significant influence on follicular development and cellular survival. Notably, OCT4 is involved in regulating multiple signaling pathways, including PI3K/AKT and GSK3β/β-catenin [10, 40]. Our findings show a significant increase in the transcription levels of OCT4 as follicle diameter increases, peaking in large follicles compared to controls and other tested follicles, thereby supporting prior studies on the role of OCT4 in follicular development.

Under normal physiological conditions, ovarian follicular cells contain antioxidant enzymes that help maintain the balance between antioxidants and oxidative systems within the ovary. This balance ensures that reactive oxygen species (ROS) levels remain within functional limits [41]. However, any disruption in the regulation of intracellular ROS levels can negatively affect oocyte function and follicular health [41, 42]. These detrimental effects have been shown to be alleviated with antioxidant treatments and synthetic activators, particularly in ART applications [43, 44]. The critical roles of catalase (CAT), superoxide dismutase (SOD), and glutathione peroxidase (GPx) in the neutralization of various oxidants and the reduction of ROS levels have been well-documented in prior studies [45].

In this study, we evaluated oxidative stress levels in ovarian follicles and observed a higher abundance of (ROS) and (MDA) levels in preantral follicles. Notably, these markers decreased as follicle size increased. Furthermore, the concentrations of key antioxidant enzymes, including (CAT), (GPx), and (SOD) were modulated across different follicular developmental stages. The large follicles exhibited the highest enzymatic activity for GPx while showing the lowest concentrations of SOD and CAT. These antioxidants markers have been previously detected in follicular fluid of various mammalian species while the activity of SOD has been shown in one report to be negatively correlated with follicle size (9), while in older women, the total CAT and SOD activity was decreased, suggesting the linkage between antioxidant activity and aging-related follicle depletion [41]. The results that also support our above-mentioned results of ROS decrease in large follicle. These findings are also consistent with our previous reports on buffalo, where we noted an increase in MDA levels under oxidative stress and in inactive ovaries, accompanied by a decrease in SOD and catalase levels [46]. Also, the decrease in oxidative stress markers was reported in healthy and growing follicles and the antioxidant defense mechanisms were modulated in response to the developmental stages [47]. Overall, our results underscore the interplay between growth markers and antioxidant activity in the regulation of buffalo folliculogenesis and underscore species-specific differences in the regulation and response of buffalo ovarian follicles. These insights could lead to the development of more effective, species-adapted IVF protocols, enhancing the reproductive efficiency of buffalo.

One of the limitations of the current work is the lack of complementary translation levels analysis, as well as the genetic functional assays including gene knockdown/overexpression for more insight into the growth markers interaction during folliculogenesis. Future work could validate the mRNA expression results at protein levels to quantify specific translational variability, as well as studying gene editing strategies during different stages of follicle development.

## Conclusion

Our study highlights the dynamic interplay between key regulatory genes and oxidative stress markers during follicular development. OCT4 expression remained stable in preantral and small follicles but was significantly upregulated in medium and large follicles, indicating its role in follicular maturation. The high expression of GDF9 and BMP15 in small follicles underscores their critical function in early follicle growth and oocyte development. FGFR2 expression increased progressively with follicle size, suggesting its involvement in later-stage follicular progression. Oxidative stress markers exhibited an inverse relationship with follicle size, as ROS, MDA, and CAT concentrations declined in larger follicles, while GPx activity increased, reflecting a shift toward an enhanced antioxidative environment. These findings suggest that as follicles mature, they develop more effective antioxidant defense mechanisms to protect oocytes from oxidative damage. Understanding these molecular and oxidative mechanisms provides valuable insights into follicle growth regulation, offering potential applications in reproductive medicine, fertility preservation, and assisted reproductive technologies. Further research into these pathways could contribute to optimizing vitro follicle culture systems and improving fertility treatments.

## Data Availability

No datasets were generated or analysed during the current study.
